# The Role of Oxidative Stress and Autophagy in Atherosclerosis

**DOI:** 10.1155/2015/130315

**Published:** 2015-03-18

**Authors:** Ida Perrotta, Saveria Aquila

**Affiliations:** ^1^Department of Biology, Ecology and Earth Sciences (Di.B.E.S.T.), Transmission Electron Microscopy Laboratory, Centre for Microscopy and Microanalysis (CM2), University of Calabria, 87036 Rende, Italy; ^2^Department of Pharmacy and Sciences of Health and Nutrition, University of Calabria, 87036 Rende, Italy

## Abstract

Atherosclerosis is a multifactorial, multistep disorder of large- and medium-sized arteries involving, in addition to age, gender and menopausal status, a complex interplay between lifestyle and genetic risk factors. Atherosclerosis usually begins with the diffusion and retention of atherogenic lipoproteins into the subendothelial space of the artery wall where they become oxidized by local enzymes and accumulate, leading to the formation of a cushion called atheroma or atheromatous or fibrofatty plaque, composed of a mixture of macrophages, lymphocytes, smooth muscle cells (SMCs), cholesterol cleft, necrotic debris, and lipid-laden foam cells. The pathogenesis of atherosclerosis still remains incompletely understood but emerging evidence suggests that it may involve multiple cellular events, including endothelial cell (EC) dysfunction, inflammation, proliferation of vascular SMCs, matrix (ECM) alteration, and neovascularization. Actually, a growing body of evidence indicates that autophagy along with the chronic and acute overproduction of reactive oxygen species (ROS) is integral to the development and progression of the disease and may represent fruitful avenues for biological investigation and for the identification of new therapeutic targets. In this review, we give an overview of ROS and autophagy in atherosclerosis as background to understand their potential role in this vascular disease.

## 1. Introduction

Reactive oxygen (ROS) and nitrogen species (RNS) are highly reactive molecules, either endogenously produced during normal metabolism in the body or exogenously introduced by the environment. Among these classes of molecules, those deriving from ROS have great biological impact because they are endogenously produced and can damage virtually all classes of macromolecules at high concentrations [1]. ROS are metabolites of oxygen that, due to their ability to easily gain and lose electrons, are prone to participate in oxidation-reduction reactions. Mammalian cells have developed various mechanisms to limit the production of ROS, inactivate them, and repair cell damage. However, when the rate of ROS production dramatically increases and/or the antioxidant defences fail or are insufficient, oxidative stress occurs [2, 3]. Oxidative stress has been demonstrated to play an important role in the pathogenesis of atherosclerosis especially by promoting the oxidative modification of low-density lipoprotein (LDL) [4]. Oxidation of LDL is one of the earliest events in atherogenesis and NADPH oxidase has been demonstrated to be critically involved in this process in both mice and humans by acting either directly or indirectly as a precursor of ROS which are utilized as substrates by other enzymes to generate more powerful oxidizing species [5–7]. ROS have been also copiously reported as early inducers of autophagy; however, to date, it is still unclear how these species exactly drive the process [8]. Autophagy is an evolutionarily conserved pathway for bulk degradation that plays critical roles in eliminating long-lived proteins, macromolecular aggregates, and damaged intracellular organelles [9, 10]. Autophagy-related proteins or Atgs, of which more than 30 have currently been identified, organize into functional complexes that oversee the autophagic process. First, Atgs concentrate on single lipid bilayer membranes (“limiting membranes” or “phagophores”) that bud from preexisting organelles such as the endoplasmic reticulum (ER) and modulate membrane elongation to form cup-shaped structures that engulf cytoplasmic components generating spherical autophagosomes. The autophagosome subsequently fuses with a preexisting lysosome and its cargo is degraded and recycled [11–13]. Through autophagy, cells rapidly degrade the old or burned-out components and generate an internal nutrient pool of macromolecules needed to sustain metabolic reactions under various environmental stresses [14]. Autophagy has also been shown to be directly involved in lipid homeostasis [15, 16]. This type of autophagy, called lipophagy, was first demonstrated in the liver and has now become a subject of intense research interest with potentially profound implications for the treatment of the diseases associated with dyslipidemias, such as diabetes and atherosclerosis [17, 18].

## 2. NADPH Oxidase as the Major Culprit of Oxidative Stress in Atherosclerosis

The key initiating event in atherogenesis is actually considered the disruption of endothelial cell homeostasis which upsets the balance between vasoconstriction and vasodilation and initiates an inflammatory tissue cascade both directly, by promoting the infiltration of inflammatory cells into the vessel wall, and indirectly, through the induction of cytokines and other inflammatory mediators, that ultimately leads to the structural and functional manifestations of the disease [19, 20]. Intrinsic to lesion formation is oxidative stress, due to the overproduction of ROS by both ECs and SMCs that are capable of generating oxidants from a variety of enzymatic systems [21]. The major sources of ROS in the vasculature are the reduced nicotinamide adenine dinucleotide phosphate (NADPH) oxidases (Nox), a group of plasma membrane-associated enzymes expressed in a variety of cells of mesodermal origin [22, 23].

NADPH oxidases were first described in the membrane of “professional” phagocytic cells of the immune system, where the high levels of ROS actively participate in host defense mediating the killing of ingested pathogens [24]. More recently, scientists have documented the presence of NADPH oxidase homologues in nonphagocytic cells, including ECs, SMCs, cardiac fibroblasts, and cardiomyocytes [25]. Under physiologic conditions, nonphagocytic NADPH oxidases have very low expression levels and ROS derived from their activity serve as second messengers in cell signalling [26]. However, upon exposure to mitogenic and/or transforming growth factors, high glucose, and hyperlipidemia, NADPH oxidases become upregulated and markedly increase ROS production [27].

Up to now a total of seven Nox homologues have been identified in humans [28]. Four are found in the vasculature (Nox1, Nox2, Nox4, and, most recently, Nox5), all with different distributions, intracellular compartmentalization, subunit compositions, and control mechanisms and thus with distinct pathophysiological functions [29]. Nox1, that is inactive under basal conditions, is expressed primarily in SMCs and at very low levels in ECs. Nox2 is present in ECs, adventitial fibroblasts, and invading inflammatory cells of developing atherosclerotic lesions. Nox4 is expressed at high levels under physiological conditions in all of the constitutive cell types of the blood vessel walls (ECs, SMCs, and adventitial fibroblasts), while Nox5, the most recent of the conventional Nox enzymes to be identified in humans, has been shown to be upregulated in atherosclerotic blood vessels and has been reported to be implicated in both EC and SMC proliferation [30, 31]. Unlike Nox1 to 4, Nox5 is calcium-dependent and does not require other subunits for its activation [32]. Several lines of evidence suggest that multiple NADPH oxidases are likely involved in a number of vascular pathologies, including Angiotensin II- (AngII-) induced hypertension and hypertrophy, serum-induced proliferation and platelet derived growth factor- (PDGF-) induced migration in SMCs, abnormal vascular growth and inflammation, and atherosclerosis [23, 33, 34]. In the context of atherosclerosis, the role of specific NADPH oxidase subunits has been investigated principally using the ApoE^−/−^ mice, the most widely studied animal model of the disease. These mice are hypercholesterolemic and spontaneously develop atherosclerotic plaques along their aorta and major arterial branches thereof. Among all the NADPH members, Nox1 has received the most attention mainly because it can be activated and regulated by many physiological and pathological stimuli, such as AngII and PDGF. It is assumed, although not yet documented in human lesions, that elevated Nox1 expression might have an active role in the pathogenesis of atherosclerosis [35, 36]. For instance, loss of Nox1 has been demonstrated to protect mice from medial hypertrophy and to decrease blood pressure while its overexpression seems to elicit opposite effects [33, 37]. The amount of collagen in the neointimal space has been also reported to be greater in mice deficient in both apolipoprotein E and Nox1 [ApoE^(−/−)^ Nox1^(−/y)^] compared to the ApoE-null animals [38]. This higher content of collagen in the intima of ApoE^−/−^  Nox1^−/y^ animals might probably reflect a reduction in the activity of matrix degrading enzymes induced by ROS. A significant reduction of macrophage accumulation and chemoattractant gene expression has been also reported in the atherosclerotic lesions of ApoE^−/−^ mice with deletion of Nox1 [35]. Besides, Nox1 has been also demonstrated to participate in the activation of SMCs since their migration and proliferation rate* in vitro* appear to be Nox1 dependent and deficiency of Nox1 is able to reduce neointimal hyperplasia following vascular injury [34, 39–41]. In summary, Nox1 may contribute to the development and progression of atherosclerotic lesions by modulating various pathways such as macrophage infiltration, cell proliferation, collagen synthesis, and lesion size.

Like Nox1, Nox2 is expressed at low physiological levels in the vasculature but becomes upregulated in cardiovascular risk settings including hypertension, diabetes, and hyperlipidemia [42–45]. Only few studies have investigated the physiopathological role of Nox2 in the vasculature most often with mixed results. Recently, Nox2 has been implicated in atherosclerosis in both humans and animal models. The first direct evidence for a role of Nox2 in atherogenesis has been provided by Judkins and coworkers in 2009 who demonstrated that Nox2 expression is upregulated in the aortic endothelium and macrophages of ApoE deficient mice before the morphological appearance of the lesion and that these changes are temporally associated with an increase of superoxide anion generation [46]. The absence of Nox2 has been also reported to inhibit the aortic production of ROS, to enhance NO bioavailability, and to markedly reduce plaque formation. It has been shown that an endothelial-specific increase in Nox2-derived superoxide production is sufficient to alter macrophage recruitment and endothelial cell activation, key factors in the initiation of atherosclerosis in ApoE^−/−^ mice [47]. Moreover, Nox2 has been shown to be highly expressed in paraffin-embedded tissue sections from coronary arteries with atherosclerosis [48]. As with Nox2, Nox4 protein has been reported to be substantially upregulated in human atherosclerotic tissues [49, 50]. However, in models for experimental atherosclerosis research such as genetically susceptible mice and primate, Nox4 protein content in aortic vessels has been found to remain stable [46, 51]. Nox4 is constitutively present in all the vascular cells where it is significantly more abundant than other Nox isoforms. The current knowledge regarding the involvement of Nox4 in atherosclerosis mostly derives from interventions in cell culture [49]. Nox4 has been shown to regulate adipogenesis by mediating preadipocyte differentiation and to modulate SMC migration* in vitro*, supporting the idea that it may be involved in the phenotypic modulation of vascular cells during atherogenesis [52, 53].

**Figure 1:**
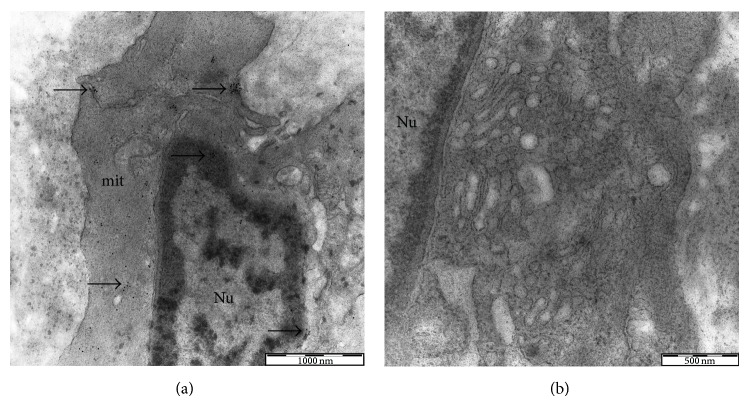
Electron microscopy immunogold labeling for NOX4 in atherosclerotic human aorta. Contractile SMCs contain numerous myofilaments and few cytoplasmic organelles (a). Nox4 immunopositivity can be observed in both the nucleus and the cytoplasm. Synthetic SMCs exhibit few myofilaments, a prominent Golgi apparatus, and numerous circular vesicles (b). Synthetic SMCs show complete absence of immunolabeling for Nox4.

In line with the* in vitro* findings, current independent evidence indicates a possible role for Nox4 in the maintenance of the differentiated state of SMCs [49, 54–57]. In this regard, our previous study demonstrated that Nox4 expression profiles differ significantly between healthy and atherosclerotic aortic tissues. Our immunocytochemical and ultrastructural analysis evidenced a strong expression of Nox4 in the contractile SMCs of both diseased and normal tissue that becomes substantially attenuated in the dedifferentiated SMCs of atheromatous plaque which display a myofibroblastic appearance (Figure 1) [58]. In some cases, Nox4 has been reported to provide significant protection against the onset of the disease. For instance, in cultured ECs, Nox4 has been found to be upregulated in response to physiological shear stress and downregulated following pathological stimuli while in the SMCs, and overexpression of Nox4 has been demonstrated to promote redifferentiation after vascular injury [23, 59–61]. Abrogation of Nox4 has been also shown to inhibit monocyte chemoattractant protein-1 and low-density lipoprotein receptor expression and ROS production in ECs exposed to oxidized phospholipids [62, 63]. The localization and the expression profile of Nox5 in human plaque appear highly dynamic. While in early lesions Nox5 appears to be exclusively expressed in ECs, in advanced lesions, the endothelial staining becomes less evident, and a large amount of protein colocalizes with the SMCs of the subintimal space [64]. Higher levels of Nox5 protein have been also documented in the SMCs adjacent to the lesion [65]. Recent studies indicate that Nox5 can directly activate eNOS in human aortic ECs leading to the production of peroxynitrite and therefore contributing to endothelial dysfunction [66]. It should be noted, however, that not only the vascular NADPH oxidase, but also the phagocytic oxidases may play an important role in the production of ROS as monocytes and lymphocytes infiltrate vascular tissues and promote functional and structural alterations. Relevant to this, it has been reported that within the lesion the activated monocytes are the only cells capable of oxidizing LDL in the absence of added free metal ions through a reaction that is entirely dependent on the production of superoxide anion by the NADPH oxidase [67–69]. In this scenario, it is likely that NADPH oxidase activity may directly participate in lipid and lipoprotein oxidation, leading to foam cell formation or may serve as a precursor for mediating myeloperoxidase and ceruloplasmin oxidation of lipids. NADPH oxidase system may also impact gene expression and vascular cell function and behavior, significantly contributing to lesion development [70].

## 3. Self-Eating in Atherosclerosis: Protective or Detrimental?

In mammals, autophagy has been implicated in the pathogenesis of a wide variety of conditions, including neurodegenerative disorders, bacterial and viral infections, cancer, and more recently atherosclerosis [71–75]. However, although many studies have been performed in animal models of atherosclerosis, the degree to which autophagy occurs in human lesion, which cells in the lesion contribute to this process, and whether autophagy impacts plaque formation are unknown. The role of autophagy in atherosclerosis seems to be complex, with reports indicating both detrimental and protective effects at the site of injury. Because autophagy is well recognized as a survival mechanism and not as a death pathway, it is tempting to assume that it might play a protective role during the development of the disease. For instance, it has been demonstrated that autophagy takes part in the defense mechanisms against oxidative stress and occurs mainly to eradicate damaged proteins and polarized mitochondria, prior to cytochrome c release and caspase activation [76]. In this way, successful autophagy can contribute to cellular recovery in the inflammatory and prooxidant milieu of the plaque, preventing SMCs apoptosis and stabilizing the lesion [77, 78].

The protective role of autophagy in atherosclerosis has been demonstrated in several* in vitro* systems. It has been shown that in cultured SMCs statin-induced cell death is partially rescued after treatment with 7-ketocholesterol (a well-known autophagy inducer). Similarly, the beneficial effects of verapamil in controlling neointima formation following vascular injury have been proved to be directly associated with the onset of autophagy [79, 80]. The autophagic machinery also plays an important role in defending ECs from AGEs and oxLDL-induced cytotoxicity [81]. In 2012, three different reports published in Cell Metabolism on genetically engineered mouse models of atherosclerosis have conclusively demonstrated the cytoprotective role of autophagy* in vivo*. In this series of studies, knockdown of an essential autophagy gene (Atg5) has been shown to exert potent proatherosclerotic actions by inducing inflammasome activation and apoptosis, subendothelial formation of cholesterol crystal, defective or inefficient efferocytosis, and impaired cholesterol efflux from macrophages [82, 83]. Accordingly, deletion of Wip1 phosphatase, a mTOR dependent inhibitor of autophagy, has been demonstrated to positively modulate lipid metabolism, prevent diet-induced obesity, and reduce the development of atherosclerotic plaque [84].

Apart from its protective activities, autophagy may also play a detrimental role in plaque formation. For instance, excessively stimulated autophagy is capable of destroying a major proportion of the cytosol and organelles finally leading to ECs and/or SMCs death (autophagic death), plaque destabilization, and acute clinical events [80, 85, 86]. Another aspect to be considered is the potential role and impact of lipophagy in the atherosclerotic artery walls. The term “lipophagy” refers to the clearance of lipid droplets in the macrophage- and SM-derived foam cells of the plaque by stimulating autophagy. Lipid droplets are intracellular storage deposits of triacylglycerols and sterol esters enclosed by a polar monolayer membrane. Mobilization of lipids inside the droplets occurs when their storage becomes too large mainly through lipolysis [18, 87]. Recently, it has been suggested that lipophagy may significantly contribute to macrophage cholesterol efflux by moving cytoplasmic lipid to lysosomes for degradation. It has been demonstrated that while normal macrophages preloaded with modified LDL can rapidly hydrolyze and reesterify them into lipid droplets, autophagy-deficient macrophages exposed to the same conditions and injected into normal mice display a reduced capacity to handle and efflux cellular cholesterol [17, 88, 89]. In addition, as mentioned above, deletion of Wip1 in ApoE^−/−^ mice has been reported to impair the autophagic flux and the conversion of macrophage into foam cells, thus preventing the buildup of atherosclerotic plaque inside the arteries [84]. Taken together this evidence demonstrates that defective lipophagy not only contributes to lysosomal dysfunction but also contributes to the development of atherosclerosis indicating a distinct mechanism of action that could be targeted for more efficacious therapy. Although the protective role of autophagy in atherosclerosis has been confirmed and documented in different* in vitro* systems and experimental models of the disease, the significance of these results in humans is less clear. This lack of knowledge may probably be due to the fact that despite the development of several methods for monitoring autophagy under controlled laboratory conditions, detection of autophagy in tissue is among the least developed areas at present [13]. As a consequence, ideal methods for detection of autophagy in atherosclerotic plaques relative to the techniques possible with cells in culture do not exist. In this context, transmission electron microscopy (TEM) has represented a useful tool to identify certain features of cell death unrelated to apoptosis and necrosis but typical of autophagy, such as the formation of myelin figures, the accumulation of ubiquitinated inclusions in the cytosol, and severe vacuolization [90–92]. The presence of structures consistent with autophagosomes has been only rarely reported in human lesions but it has been well-documented in plaques from cholesterol-fed rabbits or after treatment of SMCs with oxidized lipids [78, 79, 86, 93, 94].

**Figure 2:**
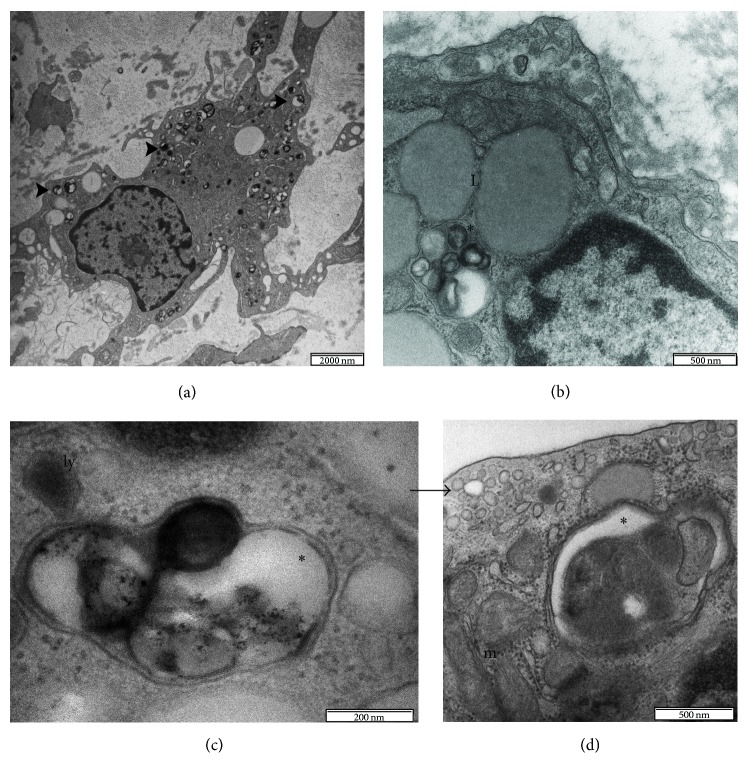
Transmission electron micrographs of atherosclerotic human aorta. Synthetic SMCs undergoing autophagy (a). Autophagic vesicles (arrowheads) generally possess a high electron density. The cytoplasmic material targeted for autophagic destruction is sequestered into a double or multilayered membranes vesicle called autophagosomes (asterisks in b, c, and d). Lipid droplets (L) are clearly distinguishable from the autophagic vesicles by virtue of their homogeneous gray appearance. Autophagosomes fuse with lysosomes (ly) and their cargo is degraded and recycled. Lysosomes usually appear as small electron-dense, single-membrane spherical vacuoles (c). The black arrow indicates an endosome; m: mitochondria (d).

Lately, our own study has demonstrated the presence of autophagy in human atherosclerotic aortas using a morphological approach based on the detection of the autophagic bodies also providing the first complete ultrastructural documentation of the autophagic process in the plaque (Figure 2) [95]. Immunoblot and immunohistochemical analyses have been also frequently employed to detect autophagy in atherosclerotic lesions although often with contradictory results [79, 86]. For instance, LC3 gene expression has been shown to be significantly decreased in the peripheral leucocytes of patients with coronary disease and increased in the lysates from advanced human plaques and in cultured SMCs under normal physiological conditions. This suggests that induction/inhibition of autophagy might not only play a role during the progression of the disease but also act as an important housekeeping mechanism to remove abnormal proteins and other cytoplasmic macromolecules or organelles even under normal physiological conditions [79, 86, 96]. Detection of granular cytoplasmic ubiquitin inclusions by immunohistochemistry represents another attractive technique, used by many groups, to assess the presence of autophagy in the cardiovascular system. In this regard, dying SMCs in the fibrous cap of advanced human lesions have been demonstrated to possess numerous ubiquitinated inclusions in their cytoplasm evocative of autophagic machinery induction [79, 97]. To date, however, many questions regarding the detection of macrophages autophagy in the plaque still remain problematic and unresolved. Normally, macrophages possess a strong phagocytic activity, which makes it difficult, if not impossible, to determine morphologically whether their vacuoles result from an autophagocytic or heterophagocytic pathway of cytoplasmic degradation [75, 85, 86]. In addition, the physiological presence of elevated levels of lysosomal hydrolases (such as cathepsin D and cathepsin L that are often used to detect autophagy in other human diseases) in the macrophages can produce false-positive results during the immunohistochemistry staining procedures [98]. Similar problems also exist for autophagy gene Beclin-1, a component of the class III PI3K complex that is required for autophagy. Beclin-1 has been extensively used to detect autophagic death in neurons however, the protein that appears to be highly expressed in normal macrophages* in vitro*; it is not differentially expressed in human plaques [98–100]. Therefore, due to the technical limitations and because of the fact that autophagy remains among the most understudied areas in cardiovascular research, the occurrence and the distribution of this process in atherosclerotic human lesions are often unappreciated and the research still offers many questions and objectives to explore, understand, describe, and explain.

## 4. Crosstalk between Oxidative Stress and Autophagy in Atherosclerosis: A Brief Overview

The involvement of oxidative stress in atherosclerosis development is well established. As already mentioned above, oxidative stress can be defined as an excessive amount of oxygen radicals inside and/or outside the cell, which is the net result of an imbalance between the production and neutralization of ROS. An increasing number of studies have consistently demonstrated that oxidative stress is of major importance in atherogenesis, especially for its participation in the oxidation of LDL [4]. Indeed, whereas native LDL does not cause cholesterol ester accumulation in macrophages and SMCs, oxLDL are cytotoxic to vascular cells and can be easily taken up by scavenger receptors resulting in foam cell formation [101]. Generally, autophagy response to ox-LDL in vascular tissue is most likely a mechanism of cell survival that protects them from dying. In this regard, several lines of evidence have demonstrated that in cultured ECs (Human Umbilical Vein Endothelial Cells, HUVECs) autophagy machinery becomes activated upon treatment with ox-LDL and that this activation contributes to the degradation of ox-LDL allowing cells to survive during harsh conditions [77]. Exposure of vascular SMCs to relatively modest concentrations of oxLDL has been also shown to trigger autophagy, whereas high levels of oxLDL suppress autophagy leading to increased apoptosis [102]. In analogy with oxLDL, the autophagy inducer 7-ketocholesterol (7-KC) appears to promote a protective form of autophagy in vascular SMCs. In this regard, it has been demonstrated that treatment of cultured SMCs with 7-KC is able to induce progressively protein dysfunctions and damage and to stimulate extensive autophagic vacuoles formation and LC3-II accumulation [80]. Administration of 7-KC to SMCs has been also demonstrated to increase protein ubiquitination and to significantly repress the cell death processes induced by low concentrations of statins [103]. Apart from ox-LDL, oxidative modification of infiltrated lipid can generate a wide variety of bioactive intermediates and end-products such as malondialdehyde, 4-hydroxynonenal (4-HNE), or 1-palmitoyl-2-oxovaleroyl phosphatidylcholine (POVPC). It has been reported that exposure of vascular SMCs to 4-HNE can induce modification and accumulation of several proteins, which must be removed to prevent further toxicity [104]. Within SMCs, the 4-HNE-modified proteins are gradually removed by autophagy as demonstrated by the fact that 4-HNE-treated cells display extensive vacuolization, phagophore formation, multilamellar structures, and pinocytosis [105]. However, the mechanisms by which 4-HNE or 4-HNE protein adducts trigger autophagy still remain unclear. It is obvious that autophagy can protect to some extent vascular cells against apoptotic cell death, depending on the cell type, oxidant concentration, and time of exposure. For instance, under conditions of severe oxidative stress, the autophagic flux is impaired and becomes unable to adequately clear misfolded proteins and superfluous or damaged organelles. High levels and/or chronic exposure to ROS can also directly disrupt the lysosomal membrane structure inducing the release of lysosomal enzyme into the cytosol and, as a consequence, the activation of the caspase pathway [76, 106]. Autophagy in atherosclerosis is also involved in the formation of ceroid, an insoluble complex of oxidized lipid and protein, which is commonly observed in human atherosclerotic lesions [75, 78]. In these concerns, it has been demonstrated that ceroid colocalizes with either macrophage-derived foam cells or SMCs in advanced plaques and also that lesional cells usually contain a large number of lysosomal ceroid deposits that impair autophagy and induce apoptosis [13, 86].

## 5. Concluding Remarks

Several lines of evidence indicate that ROS are the upstream modulators of autophagy and that oxidative stress coupled with defective autophagy may play a fundamental role in regulating atherosclerotic plaque development. The general consensus is that basal autophagy can protect plaque cells against oxidative stress by degrading damaged intracellular material and promoting cell survival. In contrast to basal autophagy, excessive stimulation of autophagy in SMCs and/or ECs may cause autophagic cell death, leading to reduced synthesis of collagen, thinning of the fibrous cap, plaque destabilization, lesional thrombosis, and acute clinical events. However, despite all the valuable knowledge gained in recent years, further work is still necessary to determine the importance of this phenomenon in human atherosclerotic plaque and to identify the critical regulatory networks that could serve as targets for preventive and therapeutic interventions. We need to understand how ROS and autophagy are induced in atherosclerotic lesions, the regulation processes governing their crosstalk and their mechanism of action, and how they could influence the biology of the plaque.
